# Case Report: Multiple cerebral infarctions in a patient with hereditary hemorrhagic telangiectasia following a fall

**DOI:** 10.3389/fgene.2025.1581625

**Published:** 2025-05-26

**Authors:** Linting Gu, Sheng Chen, Wenwei Li, Fanlong Ye

**Affiliations:** Department of Neurology, Shanghai Public Health Clinical Center, Fudan University, Shanghai, China

**Keywords:** hereditary hemorrhagic telangiectasias, stroke, gene, arteriovenous malformation, endoglin, case report

## Abstract

Hereditary hemorrhagic telangiectasia (HHT), also known as Rendu-Osler-Weber syndrome, is an autosomal dominant disorder characterized by arteriovenous malformations (AVMs) affecting multiple organs. This case report presents a rare case of a 62-year-old female with multiple cerebral infarctions following a fall, subsequently diagnosed with HHT. Clinical features included recurrent epistaxis, tongue telangiectasias, and pulmonary AVMs (PAVMs). Genetic testing identified a novel duplication mutation in the ENG gene, c.680_687dupACTCGGCC (p.G230Tfs*8). Brain MRI revealed multiple unusual infarctions, with SWI findings indicating cerebral microvascular abnormalities. These findings highlight the potential role of chronic hypoperfusion and hemodynamic dysregulation, in addition to paradoxical embolism, in HHT-related stroke mechanisms. The patient’s management included antiplatelet therapy adjustment and recommendations for regular imaging and genetic counseling. This case underscores the importance of considering HHT in acute ischemic stroke patients with vascular abnormalities and emphasizes the need for further research into the complex pathophysiology of HHT-related strokes.

## Introduction

Hereditary hemorrhagic telangiectasia (HHT) is an autosomal dominant inherited disease. The term “HHT” was first proposed by Hanes in 1909. However, since the condition had previously been described by Rendu, Osler, and Weber, it is also known as Rendu-Osler-Weber syndrome (Porteous et al., 1992). The estimated prevalence of HHT varies significantly, with a minimum rate of approximately 1 in 10,000; however, the prevalence is higher in certain geographically isolated regions (Abdalla and Letarte, 2005). It is characterized by multiple arteriovenous malformations (AVMs) affecting the nasal, buccal, and gastrointestinal mucosa, as well as the skin microvasculature and larger vessels in internal organs, primarily the lungs, liver, and brain. These manifestations are caused by loss-of-function mutations in the ENG, ACVRL1, SMAD4, and GDF2 genes (Shovlin et al., 2020). Among these, endoglin (HHT type 1) on chromosome 9q33-34 and activin receptor-like kinase 1 (ALK1) (HHT type 2) on chromosome 12q13 are the primary pathogenic loci responsible for the majority of HHT cases (Al Tabosh et al., 2024).

In this report, we present a rare case of a patient with multiple cerebral infarctions following a fall, who was subsequently diagnosed with HHT. Genetic testing revealed a duplication mutation in the ENG gene, c.680_687dupACTCGGCC (p.G230Tfs*8). This mutation causes a frameshift, leading to a premature stop codon and resulting in a truncated protein. To our knowledge, this specific mutation has not been reported in previous literature or databases, nor has this unique pattern of ischemic stroke imaging been described in prior cases.

## Case presentation

### Patient information and clinical features

A 62-year-old female was hospitalized with bilateral upper limb weakness and dizziness after tripping over a tree branch while working in the fields. She used her hands to brace the fall, causing a neck sprain but no head trauma. Upon standing, she noticed weakness in her arms and dizziness.

**FIGURE 1:**
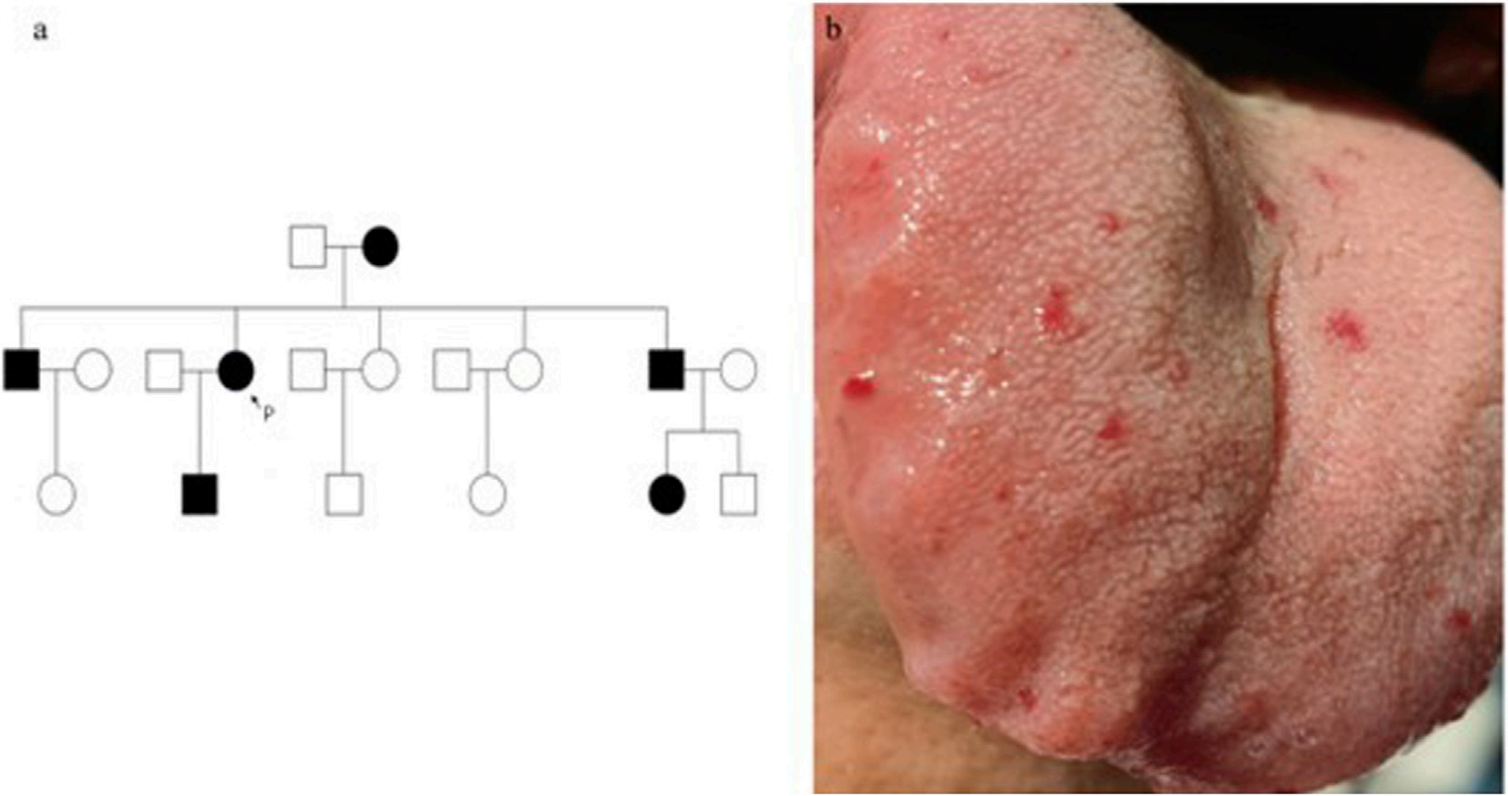
The family tree with male in □, female in ○, epistaxis in black is shown. P, proband **(a)**. Vascular lesions on the tongue. Vascular lesions on the tongue **(b)**.

Her medical and family history revealed the patient was born to non-consanguineous parents. She had frequent episodes of epistaxis since childhood, with similar symptoms reported in family members, suggesting an autosomal dominant inheritance pattern (Figure 1a).

Physical examination revealed multiple small hemangioma-like telangiectatic lesions on the tongue (Figure 1b). Neurological examination showed muscle strength in the right upper limb was 0/5, and in the left upper limb, it was 2/5. However, other neurological assessments, including cranial nerve function, sensory function, and reflexes, revealed no significant abnormalities. Vital signs were stable upon admission.

### Clinical findings

Laboratory tests rindicated slightly elevated levels of C-reactive protein (CRP), creatine kinase (CK), and D-dimer, while the complete blood count (CBC), renal function, liver function, transferrin were within normal limits. Screening for metabolic disorders, autoimmune diseases, infections, and malignancies yielded negative results.

**FIGURE 2:**
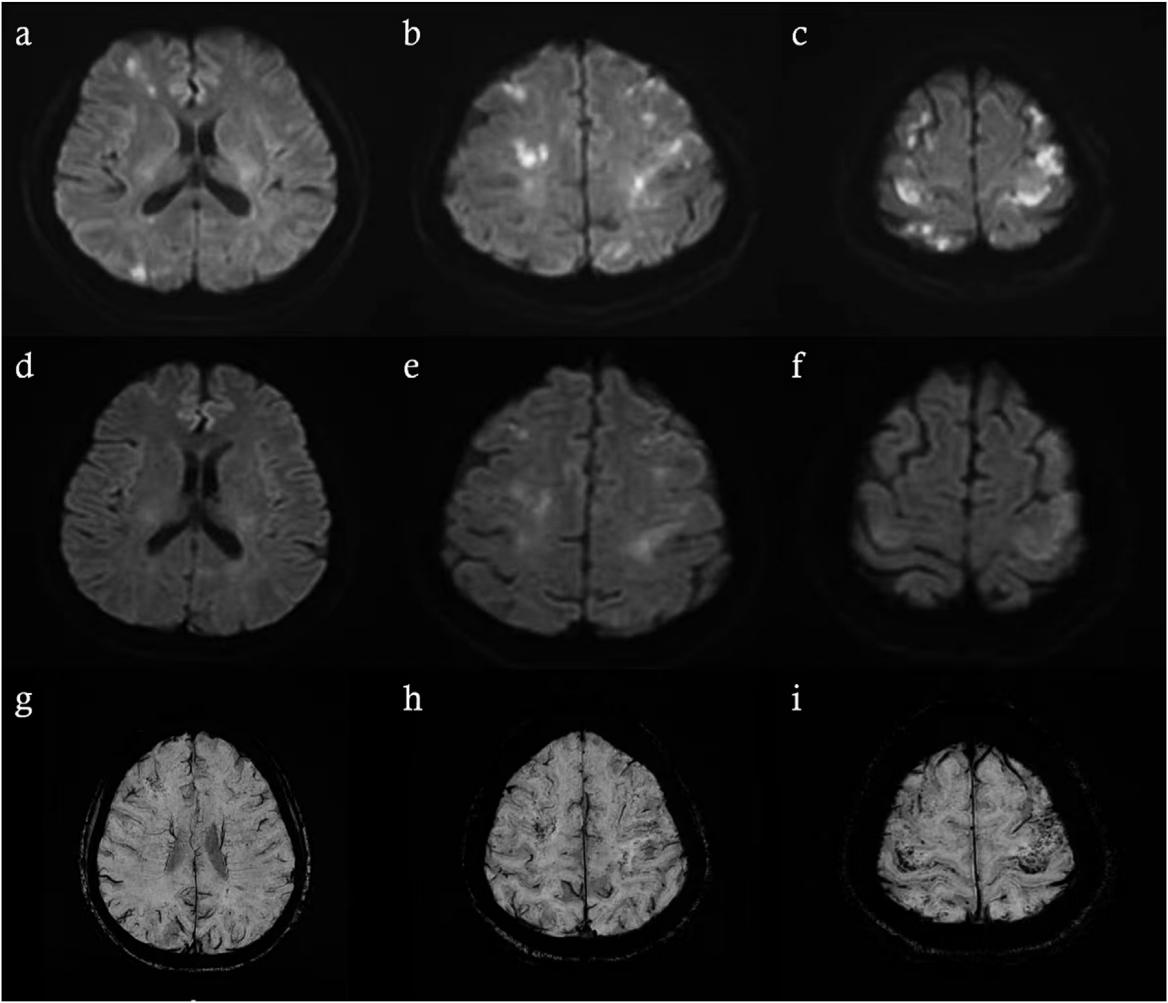
Brain MRI performed 48 h after symptom onset revealed recent infarctions **(a–c)**. Follow-up brain MRI on the 7th day of hospitalization **(d–f)**. SWI on the 7th day of hospitalization revealed scattered speckled hypointense foci in infarcted regions **(g–i)**.

**FIGURE 3:**
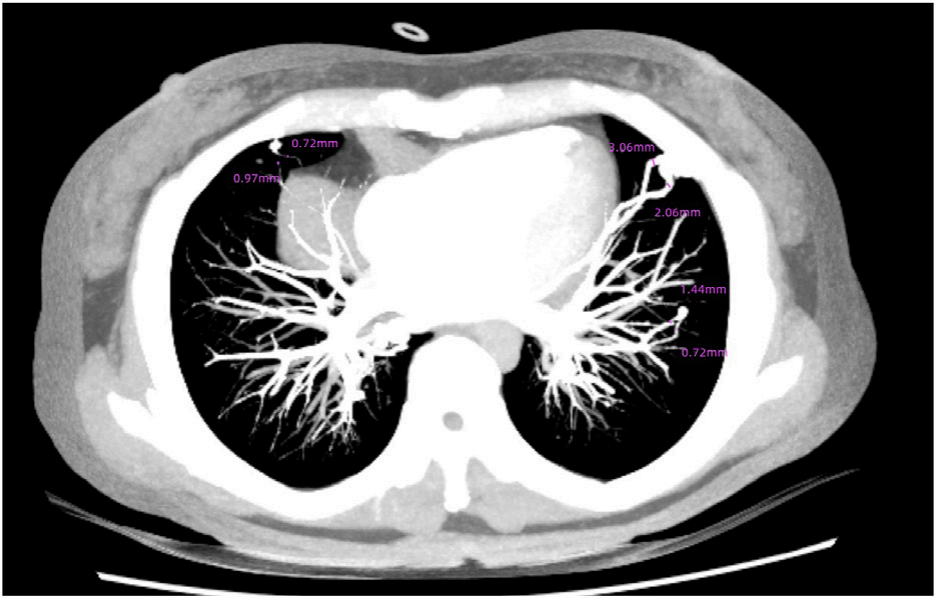
Multiple PAVMs in the left upper lobe, right middle lobe, and basal segments of both lower lobes.

Brain magnetic resonance imaging (MRI) revealed multiple infarctions in the bilateral frontal, parietal, occipital lobes, centrum semiovale, and cerebellar hemispheres (Figure 2). No abnormalities were observed in the brainstem and cervical spinal cord. Susceptibility-weighted imaging (SWI) showed capillary telangiectasias in some infarcted areas (Figure 2). Computed tomography angiography (CTA) of the head and neck revealed no significant abnormalities in the major arteries. Chest CTA showed multiple pulmonary arteriovenous malformations (PAVMs) in the left upper lobe, right middle lobe, and basal segments of both lower lobes, with feeding artery diameters ranging from 0.72 mm to 3.06 mm (Figure 3).

### Genetic testing

**FIGURE 4:**
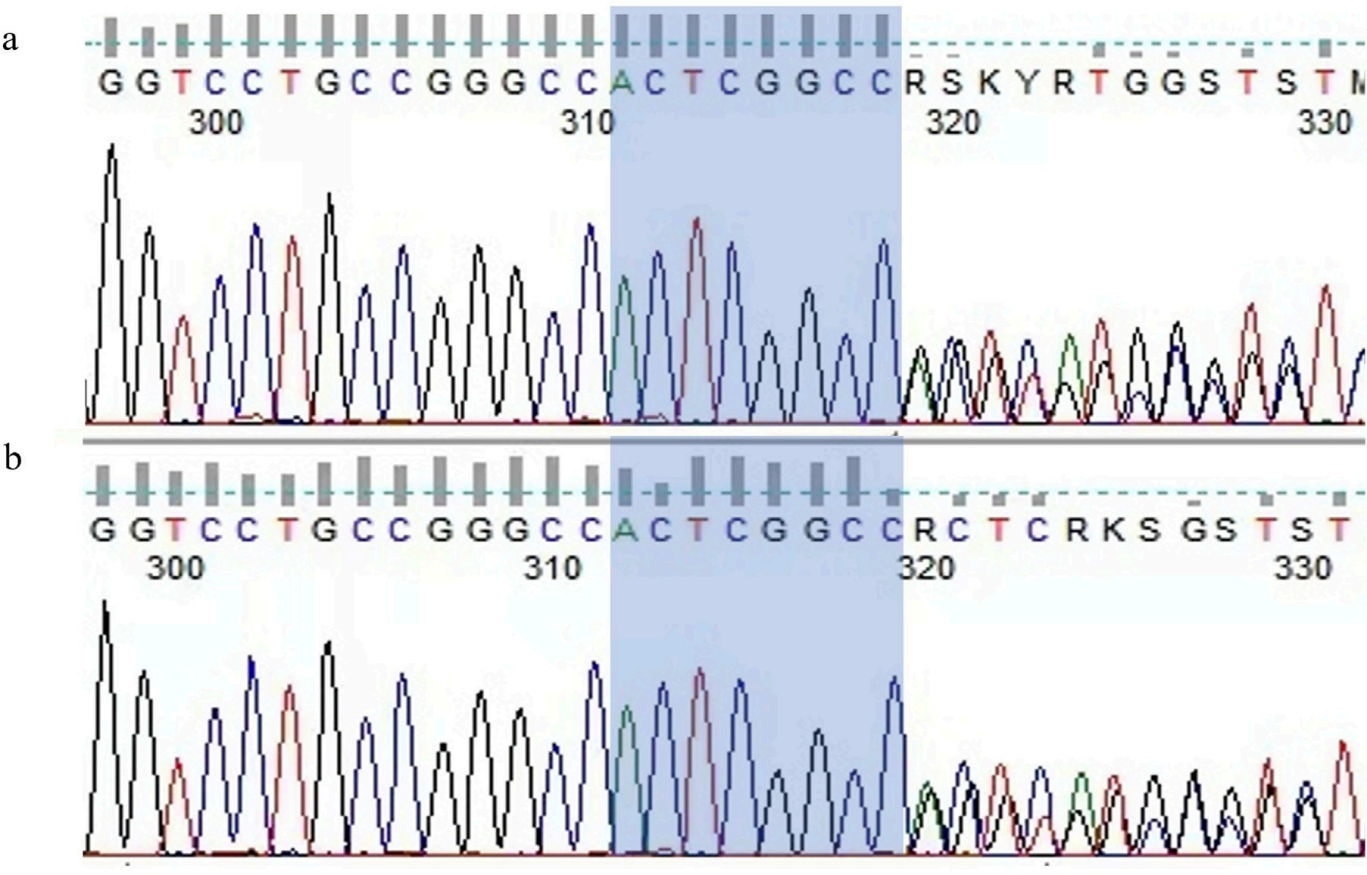
A novel mutation of ENG gene (c.680_687dupACTCGGCC) **(a,b)**.

Based on the combination of epistaxis, telangiectasia on the tongue, PAVMs and family history which were consistent with the clinical features of HHT, molecular genetic analysis was performed to confirm the diagnosis of HHT. There is a duplication mutation in the ENG gene, c.680_687dupACTCGGCC (p.G230Tfs*8) (Figure 4a). According to ACMG guidelines, the mutation of our patient has not been previously reported in the literature or genetic databases and is classified as pathogenic.

### Final diagnosis and treatment

Molecular genetic analysis identified a duplication mutation in the ENG gene, which causes a frameshift in amino acid coding and leads to premature termination. This finding confirms the diagnosis of ENG-related HHT (HHT1). Combined with the medical history and clinical examination results, the patient was ultimately diagnosed with HHT-related acute ischemic stroke. After the detection of PAVMs, embolization therapy was recommended, but the patient explicitly declined the procedure. During medical treatment, recurrent and refractory epistaxis posed a significant challenge to the management of AIS. To maintain secondary prevention while minimizing bleeding episodes, we made several adjustments to medications that might increase bleeding risk. Ultimately, the patient was maintained on clopidogrel 50 mg once daily, after which the episodes of epistaxis ceased.

### Outcome and follow-up

During hospitalization, the patient experienced no further episodes of dizziness or systemic symptoms. By day 7, the patient demonstrated full recovery of muscle strength in the left upper limb (5/5) and partial recovery in the right upper limb (proximal 4-/5, distal 5/5). At telephone follow-ups conducted at 1–2 months after discharge, the patient reported no refractory epistaxis or adverse drug reactions. After undergoing rehabilitation exercises, the patient was completely symptom-free.

As HHT is an autosomal dominant disorder, we also evaluated the patient’s younger brother, who exhibited similar symptoms. Physical examination revealed capillary telangiectasia of the tongue, and he reported a history of old cerebral infarction and hepatic hemangioma identified during previous check-ups. Due to financial constraints, further imaging studies were not performed. However, genetic testing revealed that he carried the same mutation as the patient (Figure 4b).

We recommend that all family members of this patient undergo genetic screening for HHT to enable early diagnosis and timely intervention. Family members diagnosed with HHT should also undergo comprehensive screening for vascular malformations in other organs, such as the lungs, liver, and brain, and receive appropriate treatment as early as possible.

## Discussion

Our patient presented with a rare case of ischemic stroke and a history of epistaxis, which was also reported in her family members. She additionally exhibited with telangiectasia on the tongue, and PAVMs were identified. These findings align with the diagnostic criteria for HHT, which include epistaxis, telangiectases, visceral lesions, and family history as outlined in the Curaçao criteria (Shovlin et al., 2000).

We reviewed case reports related to HHT and ischemic stroke on PubMed, excluding those without MRI findings. Ultimately, a total of six case reports (2014–2024) were included (Table 1). In these cases, ischemic strokes in HHT patients were consistently linked to PAVMs, which provide anatomical right-to-left shunts bypassing the capillary network and allowing paradoxical emboli to reach the brain (Shovlin et al., 2017).

**TABLE 1:** HHT with ischemic stroke (case reports).

Author. [Ref] Year	Age (female)	Gene	Manifestation	DWI	PAVM
Tang et al. (2024)	58(F)	ENG	Right-sided numbness	Left fronto-parietal lobe and insula	a PAVM in the right lower lung
Park et al. (2024)	50(F)	declined	Light headedness, dizziness, gait instability, occipital headaches and dyspnoea	Right cerebellum	a PAVM in the right lower lung
Lu et al. (2020)	19	declined	Progressive paralysis of the right arm and worsening of aphasia	Left frontal, temporal lobe	a PAVM in the left lower lung
Topiwala et al. (2020)	43(F)	GDF2	Postcoital right-arm numbness, weakness and transcortical motor aphasia	Left frontoparietal and insular cortices	A PAVM in the medial right upper lobe
Arai et al. (2020)	59(F)	Not mentioned	Weakness of the left half body	Right insular cortex and temporal lobe	PAVMs in the bilateral lung fields
Saji et al. (2015)	61	ENG	Behavioral disorders	Right occipito-temporal lobe	-Resection of the lower lobe was performed at the age of 28 -Recurrence of PAVM at the left lower lobe

Several risk factors for PAVM-related ischemic stroke have been identified, including the proportion of cardiac output shunted through PAVMs, hypoxemia, and iron deficiency (Brusgaard et al., 2014; Shovlin, 2014; Topiwala et al., 2021). However, there are conflicting findings in the literature regarding the significance of feeding artery diameter (Topiwala et al., 2022). Although an assessment of the relevant risk factors was performed, the evaluation of iron metabolism was insufficient, as only hemoglobin, mean corpuscular volume, and transferrin levels were measured.

Notably, our case presented with multiple bilateral cerebral infarcts, a manifestation that has been rarely reported in previous HHT cases. This unusual presentation suggests the possibility of an alternative hemodynamic mechanism underlying the patient’s condition.

Perfusion imaging plays a crucial role in the assessment of cerebral hemodynamics. Techniques such as CT perfusion (CTP) and MR perfusion (PWI/ASL) provide both quantitative and qualitative evaluations of cerebral blood flow (CBF), cerebral blood volume (CBV), and mean transit time (MTT). These parameters are valuable for detecting chronic hypoperfusion that may be missed by conventional structural imaging and for differentiating reversible penumbral tissue from irreversibly infarcted areas during the acute phase of stroke. Although perfusion imaging was not performed in this patient due to personal reasons, we recommend its inclusion in the evaluation of HHT patients with cerebral infarction, as it can elucidate the effects of vascular malformations on both local and global brain perfusion and help identify regions at risk due to chronic microvascular dysfunction or shunting.

In this case, we utilized SWI as an additional modality. SWI is a highly sensitive MRI sequence capable of detecting subtle magnetic susceptibility variations within tissues. This technique leverages phase differences caused by iron-containing substances (such as deoxyhemoglobin, ferritin, and hemosiderin) to produce high-resolution visualization of small vessels, microbleeds, venous structures, and calcifications. In cerebrovascular disease, SWI is especially useful for detecting minute venous malformations, capillary telangiectasia, foci of bleeding, and iron deposition. In our patient, SWI revealed scattered punctate hypointense lesions within the infarcted area, suggesting the presence of multiple small vascular malformations and providing imaging evidence supporting the hypothesis of chronic hypoperfusion or microcirculatory disturbance. These vascular malformations disrupt normal hemodynamics and alter CBF, which is essential for oxygen and energy delivery to brain tissue and clearance of metabolic waste or toxic substances (Claassen et al., 2021), resulting in a chronic hypoperfusive state. To our knowledge, this perspective has not been described in previous HHT case reports; however, similar mechanisms have been discussed in studies of ischemic stroke associated with moyamoya disease (Ballout et al., 2023; Chen et al., 2021). In this patient, neck rotation and subsequent postural changes may have further destabilized cerebral blood flow, leading to insufficient perfusion, particularly in brain regions already compromised by chronic hypoperfusion. Such a mechanism has also been observed in certain cases of paroxysmal, posture-induced transient ischemic attack (TIA) (Bund et al., 2018; Manocha et al., 2022). This secondary insult likely led to acute decompensation and cytotoxic edema in borderzone regions. The partial resolution of DWI lesions observed on follow-up MRI at day 7 supports this hypothesis.

In conclusion, this case underscores the importance of considering HHT in patients presenting with ischemic strokes, especially a history suggestive of vascular abnormalities, such as PAVMs. The complex interplay between PAVMs and cerebral blood flow abnormalities highlights the necessity of further studies to better understand the underlying mechanisms and develop more effective strategies to prevent ischemic complications in HHT patients.

## Data Availability

The original contributions presented in the study are included in the article/supplementary material. The raw variation data reported in this paper have been uploaded to Figshare at https://doi.org/10.6084/m9.figshare.28926920.v3.
